# Impact of pathological response after neoadjuvant chemotherapy on adjuvant therapy decisions and patient outcomes in gastrointestinal cancers

**DOI:** 10.1002/cnr2.1412

**Published:** 2021-05-25

**Authors:** Sheena Bhalla, Huili Zhu, Jung‐Yi Lin, Umut Özbek, Eric J. Wilck, Sanders Chang, Xiuxu Chen, Stephen Ward, Noam Harpaz, Alexandros D. Polydorides, William Miller, Maria Isabel Fiel, Ippolito Modica, Wen Fan, Nebras Zeizafoun, Celina Ang

**Affiliations:** ^1^ Division of Hematology and Medical Oncology Icahn School of Medicine at Mount Sinai New York USA; ^2^ Department of Internal Medicine Icahn School of Medicine at Mount Sinai New York USA; ^3^ Department of Population Health Science and Policy Tisch Cancer Institute, Icahn School of Medicine at Mount Sinai New York USA; ^4^ Department of Radiology Icahn School of Medicine at Mount Sinai New York USA; ^5^ Department of Pathology Loyola University Medical Center Maywood Illinois USA; ^6^ Department of Pathology Icahn School of Medicine at Mount Sinai New York USA

**Keywords:** biomarkers, colorectal cancer, gastric cancer, neoadjuvant chemotherapy, pancreatic cancer

## Abstract

**Background:**

Neoadjuvant chemotherapy (NAC) is frequently used in gastrointestinal cancers (GIC), and pathological, radiological, and tumor marker responses are assessed during and after NAC.

**Aim:**

To evaluate the relationship between pathologic, radiologic, tumor marker responses and recurrence‐free survival (RFS), overall survival (OS), adjuvant chemotherapy (AC) decisions, and the impact of changing to a different AC regimen after poor response to NAC.

**Methods and results:**

Medical records of GIC patients treated with NAC at Mount Sinai between 1/2012 and 12/2018 were reviewed. One hundred fifty‐six patients (58.3% male, mean age 63 years) were identified. Primary tumor sites were: 43 (27.7%) pancreas, 62 (39.7%) gastroesophageal, and 51 (32.7%) colorectal. After NAC, 31 (19.9%) patients had favorable pathologic response (FPR; defined as College of American Pathologists [CAP] score 0–1). Of 107 patients with radiological data, 59 (55.1%) had an objective response, and of 113 patients with tumor marker data, 61 (54.0%) had a ≥50% reduction post NAC. FPR, but not radiographic or serological responses, was associated with improved RFS (HR 0.28; 95% CI 0.11–0.72) and OS (HR 0.13; 95% CI 0.2–0.94). Changing to a different AC regimen from initial NAC, among all patients and specifically among those with unfavorable pathological response (UPR; defined as CAP score 2–3) after NAC, was not associated with improved RFS or OS.

**Conclusions:**

GIC patients with FPR after NAC experienced significant improvements in RFS and OS. Patients with UPR did not benefit from changing AC. Prospective studies to better understand the role of pathological response in AC decisions and outcomes in GIC patients are needed.

## 1 INTRODUCTION

Over the last decade, there has been an increasing interest in the use of neoadjuvant chemotherapy (NAC) in patients with gastrointestinal cancers (GIC). NAC has emerged as an attractive option in both borderline resectable and resectable disease due to its potential benefits, including improved margin‐negative resection rates, tumor downstaging, decreased lymph node positivity, early treatment of presumed micrometastatic disease, and improved delivery and tolerance of chemotherapy that is not hindered by postoperative complications.^1^, ^2^, ^3^, ^4^, ^5^ As NAC is utilized more frequently, it is crucial to identify factors predictive of improved survival and recognize patients who are at greatest risk for recurrence.

Currently, pathological, radiological, and tumor marker responses are routinely assessed during and after neoadjuvant therapy, and our understanding of the role of pathological response, in particular, on patient outcomes is evolving. Similar to other malignancies in which achieving pathological complete response (pCR) after NAC is associated with better overall and disease‐free survival, pCR in GIC typically corresponds with improved outcomes.^6^, ^7^, ^8^ For instance, pCR after NAC has been shown to improve long‐term survival in patients with esophageal,^9^, ^10^ colon,^11^ and rectal^12^ cancers. While the rate of pCR is relatively low in pancreatic cancer, making it difficult to assess the impact on survival,^13^ studies have also reported an association with significantly prolonged survival.^14^, ^15^

On the other hand, unfavorable pathological treatment responses, including College of American Pathologists (CAP) scores 2 and 3, are frequently observed in clinical practice, yet are not well understood in GIC. Prior studies have not investigated how factors, such as tumor marker response after NAC or changing adjuvant chemotherapy (AC) from the NAC regimen, in patients with unfavorable pathological responses may influence outcomes. Though poor pathological response may intuitively suggest ineffective neoadjuvant treatment and prompt a change in the adjuvant chemotherapy regimen, this strategy has not been widely adapted as clinical trials showing efficacy of perioperative chemotherapy in GIC utilized similar regimens pre‐ and post‐surgery. Thus, with limited prospective data to date, post‐surgical systemic treatment decisions based on pathological response remain controversial and oncologist‐dependent. In this study, we aimed to assess how pathological treatment responses impacted patient outcomes and AC decisions in patients with GIC who underwent NAC followed by surgical resection.

## 2 METHODS

### 2.1 Patients

Institutional review board approval was obtained to review the medical records of consecutive patients with GIC, including pancreatic, gastroesophageal, and colorectal cancers, who received NAC followed by surgery between January 2012 and December 2018 at the Mount Sinai Hospital. Patients with biopsy proven pancreatic, gastroesophageal, and colorectal adenocarcinomas who underwent NAC and surgical resection were included. Though neoadjuvant radiation therapy (RT) was allowed after NAC, patients who underwent concurrent chemotherapy and radiation alone were excluded. Patients with squamous cell carcinoma were excluded.

### 2.2 Data collection

Demographic and clinical data including cancer stage, systemic and locoregional therapies, pathological response, radiographic and tumor marker results, recurrence, and vital status were collected. Post‐surgical specimens were assessed with CAP protocols for tumor, margin, and nodal (TNM) assessment.^16^, ^17^, ^18^ Pathological treatment response was scored according to CAP criteria: Complete Response, score 0 (no viable cancer cells); Near Complete Response, score 1 (single/rare groups of cancer cells); Partial Response, score 2 (residual cancer with regression); Poor/No response, score 3 (no tumor regression). Favorable pathological response (FPR) was defined as CAP score 0–1, and unfavorable pathological response (UPR) as score 2–3.

Baseline and pre‐surgical radiologic staging were performed in all patients, using computed tomography (CT), positron emission tomography (PET), or magnetic resonance imaging (MRI). Radiographic responses were classified according to response evaluation criteria in solid tumors (RECIST 1.1).^19^ Serial tumor marker (Cancer Antigen 19‐9 [CA 19‐9], Carcinoembryonic Antigen [CEA]) responses were evaluated pre‐ and post‐NAC. Tumor marker response was defined as ≥50% reduction in CA 19‐9 or CEA after NAC.

### 2.3 Statistical analysis

Descriptive statistics were calculated to summarize baseline characteristics, including demographics, disease characteristics, and treatment characteristics. Recurrence‐free survival (RFS) was measured from date of resection until detection of local recurrence, metastases, or death. Overall survival (OS) was measured from start date of NAC until death. Kaplan‐Meier curves were used to estimate the median follow‐up time, RFS and OS. Univariable and multivariable Cox proportional hazards models were fitted to identify predictors of RFS and OS. Variables that were significant in the univariable models were added to the multivariable models. In addition to identifying predictors of RFS and OS in the overall GIC cohort, we also performed the analyses in each of the cancer subtype cohorts. Univariable and multivariable logistic regression were fitted to identify the predictors of changing AC. *p* Values of less than .05 were considered to be statistically significant and hazard ratios and odds ratios with 95% confidence intervals were provided. Statistical analyses were performed using R 3.6.3 (Vienna, Austria).

## 3 RESULTS

### 3.1 Baseline patient characteristics and treatment regimen

A total of 156 patients were identified. The mean age was 63 years (SD 13 years), with slight male (58.3%) and White non‐hispanic (40.4%) predominance (Table 1). The majority of patients had an ECOG performance status of 0 to 1 (83.3%, *n* = 141). Primary tumor sites were: 43 (27.6%) pancreas, 62 (39.7%) gastroesophageal, and 51 (32.7%) colorectal. The cohort included 24 (15.4%) with stage 1 disease, 45 (28.9%) with stage 2 disease, 40 (25.6%) with stage 3 disease, and 47 (30.1%) with stage 4 disease (as per AJCC 8th edition TNM staging system).^20^ Patients underwent a median of 5 cycles of NAC (range, 1–16 cycles), and 24 (15.5%) patients received neoadjuvant RT. In this cohort, 115 (73.7%) patients received AC for a median of 5 cycles (range 1–33), and 48 (30.7%) switched to an AC regimen different from NAC regimen. Chemotherapy regimens are listed in Table S1.

**TABLE 1:** Baseline patient characteristics

Variable	*N* = 156 patients
Age, mean (SD)	63 (13)
Sex, *N* (%)	
Female	64 (41.3)
Male	91 (58.3)
Race/Ethnicity, *N* (%)	
White Non‐Hispanic	63 (40.4)
Black	26 (16.7)
Hispanic	29 (18.6)
Asian	29 (18.6)
Other or unavailable	9 (5.7)
Primary tumor sites, *N* (%)	
Pancreatic	43 (27.6)
Gastroesophageal	62 (39.7)
Colorectal	51 (32.7)
Clinical stage, *N* (%)	
1	24 (15.4)
2	45 (28.9)
3	40 (25.6)
4	47 (30.1)
Cycles NAC, median (range)	5 (1‐16)
Received neoadjuvant radiation, *N* (%)	24 (15.5)
Received AC, *N* (%)	115 (73.7)
Cycles AC, median (range)	5 (1‐33)
Known switch in AC from NAC regimen, *N* (%)	48 (30.7)
CAP treatment effect score, *N* (%)	
0	13 (8.3)
1	18 (11.6)
2	54 (34.6)
3	71 (45.5)
Radiological response available (*N* = 107), *N* (%)	
Complete response	9 (8.4)
Partial response	50 (46.7)
Stable response	47 (43.9)
Progressive disease	1 (0.9)
≥50% tumor marker response available (*N* = 113), *N* (%)	
Yes	61 (54.0)
No	52 (46.0)

Abbreviations: AC, adjuvant chemotherapy; NAC, neoadjuvant chemotherapy.

### 3.2 Survival outcomes

With a postoperative median follow‐up of 30.6 (95% CI 28.1–35.4) months, 66 (42.3%) patients developed recurrence of cancer. At last follow‐up, 30 (19.2%) patients had died. The median 2‐year RFS rates among pancreatic, gastroesophageal, and colorectal patients were 38, 66, and 71%, respectively, and the median 5‐year RFS rates were 32, 56, and 45%, respectively. The median 2‐year OS rates among pancreatic, gastroesophageal, and colorectal patients were 82, 84, and 92% respectively, and the median 5‐year OS rates were 28, 66, and 81%, respectively (Figure S1).

### 3.3 Predictors of recurrence and survival

After NAC, 31 (19.9%) patients had a FPR (CAP 0‐1), and 125 (80.1%) had an UPR (CAP 2‐3; Table 1). Of 107 patients with radiological data available, 59 (55.7%) demonstrated complete or partial response by RECIST 1.1 after NAC. Of 113 patients with serological data available, 61 (54.0%) had a ≥50% reduction in tumor maker levels after NAC.

In univariable analysis, FPR was associated with improved RFS (HR 0.26; 95% CI 0.10–0.64), and improved OS (HR 0.11; 95% CI 0.02–0.83), but radiographic response and ≥50% tumor marker response were not significantly associated with RFS and OS (Table 2). Additionally, given the relatively even distribution of patients with a CAP score 3 (54.5%) vs 0–2 (45.5%) in our cohort, we further analyzed the associations between these two groups. CAP score 3 was associated with decreased RFS (HR 1.89; 95% CI 1.16–3.09) and decreased OS compared to CAP scores 0–2 (HR 2.15; 95% CI 1.02–4.53). Positive surgical margins were associated with decreased RFS (HR 4.42; 95% CI 2.32–8.41) and OS (HR 5.94; 95% CI 2.42–14.50).

**TABLE 2:** Univariable analysis of factors associated with recurrence‐free and overall survival after NAC and surgery

Variable	Recurrence‐free survival		Overall survival	
	HR (95% CI)	*p*	HR (95% CI)	*p*
FPR (CAP 0‐1)	0.26 (0.10‐0.64)	0.003	0.11 (0.02‐0.83)	0.032
CAP score 3 vs 0‐2	1.89 (1.16‐3.09)	0.030	2.15 (1.02‐4.53)	0.043
Partial or complete radiographic response	1.16 (0.67‐2.03)	0.599	1.14 (0.48‐2.71)	0.765
≥50% tumor marker response	1.20 (0.67‐2.16)	0.532	0.65 (0.28‐1.54)	0.330
Clinical stage (4 vs 1‐3)	0.91 (0.54‐1.55)	0.737	0.88 (0.40‐1.91)	0.660
Pathological stage (4 vs 1‐3)	1.27 (0.75‐2.15)	0.367	1.67 (0.79‐3.51)	0.178
Positive surgical margins	4.42 (2.32‐8.41)	<0.001	5.94 (2.42‐14.5)	<0.001
Received AC	1.57 (0.80‐3.09)	0.187	0.90 (0.27‐2.97)	0.864
Change of AC				
Yes vs no change	2.21 (1.25‐3.93)	0.006	2.21 (0.87‐5.74)	0.097
No AC vs no change	1.75 (0.93‐3.29)	0.082	3.07 (1.25‐7.54)	0.015

Abbreviations: AC, adjuvant chemotherapy; CAP, College of American Pathologists; FPR, favorable pathologic response; NAC, neoadjuvant chemotherapy.

In sub‐analyses of gastroesophageal, pancreatic, and colorectal groups, FPR was not associated with RFS or OS (Tables S2 and S3). Notably, there was an association between CAP score 3 and decreased RFS (HR 3.73; 95% CI 1.37–10.15) and OS (HR 11.67; 95% CI 1.49–91.4) in the gastroesophageal cohort. Positive surgical margins were associated with decreased RFS in gastroesophageal (HR 5.98; 95% CI 2.1–17.04), pancreatic (HR 3.56; 95% CI 1.17–10.83), and colorectal (HR 4.25; 95% CI 1.18–15.30) cohorts, yet association with decreased OS was seen only in the gastroesophageal (HR 6.95; 95% CI 1.70–28.3) and colorectal (HR 25.3; 95% CI 4.10‐156.8) cohorts. Pathological stage (4 vs 1–3) was associated with decreased RFS in the gastroesophageal (HR 9.17; 95% CI 3.14–26.83) and pancreatic (HR 40.50; 95% CI 2.53–647.48) cohorts and decreased OS in the gastroesophageal cohort (HR 12.69; 95% CI 3.50–44.10), but no significant association with RFS or OS was seen in the colorectal group.

In multivariable analysis, FPR was associated with improved RFS (HR 0.28; CI 0.11–0.72) and improved OS (HR 0.13; 95% CI 0.20–0.94) when compared to UPR (Table 3). Positive surgical margins were again associated with decreased RFS (HR 4.47; 95% CI 2.3‐8.67) and OS (HR 5.43; CI 2.19–13.43). In multivariable subgroup analysis, positive surgical margins were significantly associated with decreased RFS in gastroesophageal (HR 5.90; 95% CI 1.99–17.52) and pancreatic (HR 3.90; 95% CI 1.27–11.96) groups.

**TABLE 3:** Multivariable analysis for recurrence‐free and overall survival after NAC and surgery

Variable	Recurrence‐free survival		Overall survival	
	HR (95% CI)	*p*	HR (95% CI)	*p*
FPR (CAP 0‐1)	0.28 (0.11‐0.72)	0.008	0.13 (0.02‐0.94)	0.043
Positive surgical margins	4.47 (2.30‐8.67)	<0.001	5.43 (2.19‐13.43)	<0.001
Change of AC				
Yes vs no	1.58 (0.87‐2.85)	0.132	1.51 (0.58‐3.92)	0.395
No AC vs no change	1.74 (0.92‐3.28)	0.086	2.85 (1.15‐7.03)	0.023

Abbreviations: AC, adjuvant chemotherapy; CAP, College of American Pathologists; FPR, favorable pathologic response; NAC, neoadjuvant chemotherapy.

**FIGURE 1:**
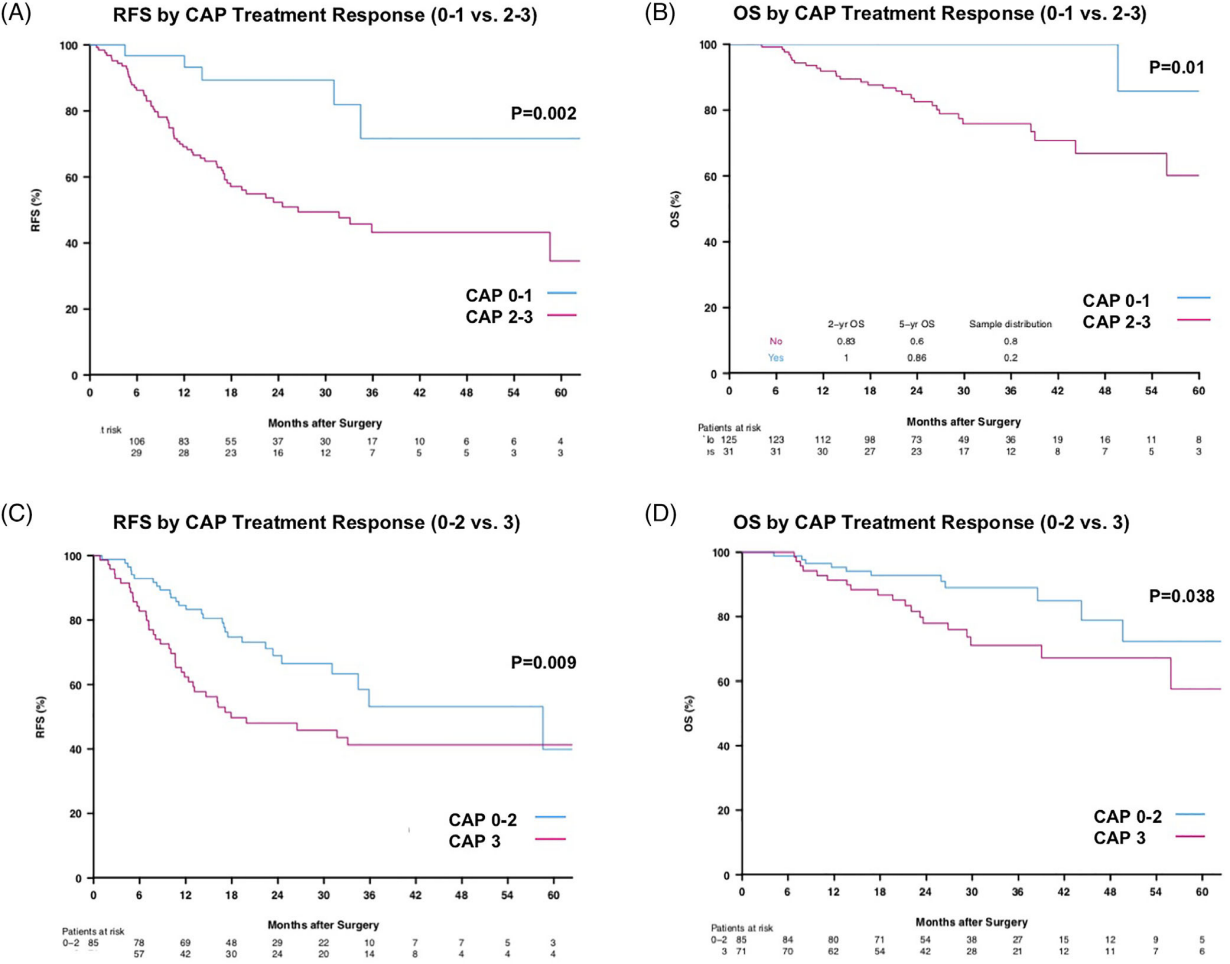
Kaplan–Meier survival curves for RFS and OS based on CAP treatment response scores. CAP, College of American Pathologists; OS, overall survival; RFS, recurrence‐free survival

**FIGURE 2:**
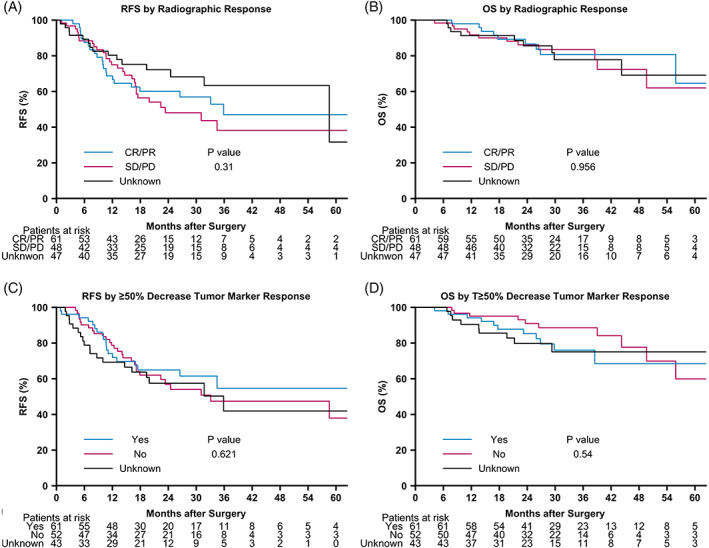
Kaplan–Meier survival curves for RFS and OS based on radiographic response by RECIST 1.1 and ≥50% tumor marker response. CAP, College of American Pathologists; CR, complete response; OS, overall survival; PD, progressive disease; PR, partial response; RFS, recurrence‐free survival; SD, stable disease

Two‐ and five‐year RFS and OS were significantly higher in patients with FPR vs UPR and in patients with CAP 0–2 vs 3 (Figure 1). In patients with available radiographic data, the 2‐ and 5‐ year RFS and OS in those with complete or partial radiographic response vs no response did not differ significantly (Figure 2A,B). Likewise, in patients with available tumor marker data, there were no significant differences in 2‐ and 5‐year RFS and OS in those with ≥50% reduction in tumor markers vs no reduction (Figure 2C,D).

In univariable analysis, receipt of AC was not significantly associated with RFS (HR 1.57; 95% CI 0.8–3.09) nor OS (HR 0.90; 95% CI 0.27–2.97) in the overall cohort (Table 2) but was associated with decreased RFS in the colorectal cohort (HR 4.78; 95% CI 1.5–15.19; Table S1). Changed AC from the NAC regimen was associated with significantly decreased RFS (HR 2.21; 95% CI 1.25–3.93), but not OS (HR 2.21; 95% CI 0.87–5.74). Patients who did not receive AC had worse OS compared to patients who received AC but did not switch regimens (HR 3.07; 95% CI 1.25–7.54).

In multivariable analysis, changing AC from the NAC regimen did not significantly impact RFS or OS, but patients who did not receive AC had significantly worse OS compared to patients who received AC but did not change regimen (HR 2.85; 95% CI 1.15–7.03).

### 3.4 Predictors of recurrence and survival in patients with a UPR

We assessed how predictive factors affected outcomes in patients with UPR. In multivariable analysis, advanced pathological stage (4 vs 0–3) and positive surgical margins were associated with reduced RFS and OS in patients with UPR (Table 4). Changing AC did not significantly affect RFS (HR 1.01; 95% CI 0.55–1.87) nor OS (HR 1.32; 95% 0.49–3.55) after adjusting for pathological stage, surgical margin, and cancer type. Again, decreased OS was noted in patients who did not receive AC compared to those who received AC but did not change regimen (HR 3.15; 95% CI 1.24–8.51). The presence of a tumor marker response did not significantly impact RFS (HR 1.22; 95% CI 0.66–2.26) nor OS (HR 0.61; 95% CI 0.25–1.47) in patients who demonstrated UPR.

**TABLE 4:** Multivariable analysis for recurrence‐free and overall survival if UPR (CAP 2–3)

Variable	Recurrence‐free survival		Overall survival	
	HR (95% CI)	*p*	HR (95% CI)	*p*
Change of AC				
Yes vs no	1.01 (0.55–1.87)	0.969	1.32 (0.49–3.55)	0.584
No AC vs no change	1.84 (0.91–3.69)	0.088	3.15 (1.24–8.05)	0.016
Pathological stage (4 vs 0‐3)	2.75 (1.82–4.15)	<0.001	1.42 (0.99–2.03)	0.056
Positive surgical margin	4.09 (2.08–8.03)	<0.001	5.24 (2.12–12.95)	<0.001

Abbreviations: AC, adjuvant chemotherapy; CAP, College of American Pathologists; UPR, unfavorable pathological response.

### 3.5 Predictors of changing adjuvant therapy

We were also interested in understanding factors associated with changing AC after NAC and surgery. The patients with FPR were less likely to have a change in AC regimen (OR 0.05; CI 0.01–0.39), while there was no significant association between AC regimen and radiographic (HR 0.76; CI 0.32–1.79) and tumor marker responses (HR 1.18; CI 0.49–2.87; Table 5).

**TABLE 5:** Univariable analysis of factors associated with changing AC after NAC and surgery

Variable	OR (95% CI)	*p*
FPR (CAP 0‐1)	0.05 (0.01–0.39)	0.004
Complete or partial radiographic response	0.76 (0.32–1.79)	0.527
≥50% tumor marker response	1.18 (0.49–2.87)	0.707
Pathological stage (4 vs 1‐3)	1.62 (1.15–2.28)	0.006
Positive surgical margin	2.69 (0.74–9.77)	0.133

Abbreviations: AC, adjuvant chemotherapy; CAP, College of American Pathologists; FPR, favorable pathologic response; NAC, neoadjuvant chemotherapy.

## 4 DISCUSSION

NAC has become increasingly common among locally advanced and resectable GIC. Although neoadjuvant therapy approaches vary based on location and stage of cancer, NAC is generally associated with potential advantages, including early treatment of micrometastatic disease, tumor downstaging, and improved margin‐negative resection rates. In patients who have received NAC, traditional pathologic features such as primary tumor size, margin status, and number of lymph nodes involved are no longer sufficient. Thus, pathological, serological, and radiographic responses are monitored, however their predictive role remains unclear and published data has been inconsistent.

In our cohort of pancreatic, gastroesophageal, and colorectal patients undergoing NAC for both borderline resectable and resectable disease, ~20% of patients demonstrated FPR. Of those with available data, 56% of patients demonstrated radiographic response, and 54% demonstrated ≥50% reduction in tumor markers. FPR was associated with significant improvement in both RFS (HR 0.28; 95% CI 0.11–0.72) and OS (HR 0.13; 95% CI 0.20–0.94), while radiographic and serological responses were not associated with these outcomes.

Though radiographic and serological responses are routinely monitored with NAC, evidence supporting their predictive role in terms of resectability and outcomes among GIC is limited. For instance, in pancreas cancer, structural imaging has significant limitations in the evaluation of treatment with chemotherapy/radiotherapy as CT and MRI cannot distinguish residual or necrotic tumor from fibrosis and radiation changes after treatment.^21^ A recent study from the Mayo Clinic suggests that complete metabolic response by PET imaging highly correlates with major pathological response among patients with pancreas cancer who underwent total neoadjuvant therapy,^22^ suggesting further evaluation of metabolic imaging is warranted in this setting. Additionally, the value of tumor marker response after NAC varies among GIC. CEA clearance pattern has proven to be independent predictor of tumor response to neoadjuvant treatment in patients with rectal cancer.^23^ In contrast, CA 19‐9 response in pancreas cancer is unlikely a sole indicator of response, though has demonstrated association with R0 resection rate, pathological response, and survival.^24^ Moreover, the optimal cut‐off point for CA 19‐9 “response” remains unknown. Prior reports suggest that a minimal decrease of 50% during NAC is associated with R0 resection rate and survival, yet other studies report that normalization of CA 19‐9 is correlated with optimal survival.^22^, ^24^, ^25^

Our study demonstrates that pathologic response predicts RFS and OS, which is also supported by literature across GI cancers. Complete pathologic response after NAC has been shown to significantly improve long‐term survival in patients with esophageal cancer^9^, ^10^ and colon cancer.^11^ Complete pathologic response in pancreatic cancer is rare,^26^ but a few studies have reported an association with significantly prolonged survival.^14^, ^15^ Patients with complete pathologic response after chemoradiation have also been reported to have better long‐term outcomes in rectal cancer.^12^, ^27^, ^28^

The majority of studies to date have focused on complete pathological response (CAP score of 0) and occasionally CAP score of 1, yet the relationship between CAP score of 2 and patient outcomes remains ambiguous, despite its frequency in the real‐world setting. We therefore assessed whether outcomes differed when CAP 2, which indicates residual tumor with regression and was present in 34% of patients in our study, was grouped with more favorable vs unfavorable pathologic responders. When CAP 2 was combined in the FPR cohort, we continued to observe significant improved survival compared to CAP 3, though this divergence appears greater with initial FPR vs UPR grouping, and on sub‐analyses, this association was noted only in the gastroesophageal cohort. These findings suggest that CAP 2 may be associated with favorable prognosis, yet this may vary by tumor type and warrants further evaluation.

The decision to change AC from the NAC regimen based on pathological, radiographic, and/or serological responses also remains controversial and oncologist dependent. In this study, FPR, but not favorable radiographic or serological responses, was associated with decreased odds of changing AC from the NAC regimen. Among all patients, changing AC was associated with decreased RFS (HR 2.21; 95% CI 1.25–3.93), however no survival benefit was noted. Among patients with UPR, changing AC did not affect RFS or OS, suggesting that patients with poor treatment effect may face poor prognosis despite switching adjuvant therapy, based on the underlying biology of their disease. Randomized controlled trials among patients with non‐GI solid tumors have explored this question with mixed results. Among patients with locally advanced breast cancer with poor pathological response after preoperative doxorubicin‐based chemotherapy, treatment with alternate non‐cross resistant chemotherapy was associated with a trend toward improved RFS and OS compared to continuing the initial chemotherapy regimen.^29^ Yet, among patients with sarcoma whose tumors showed a poor response to preoperative chemotherapy, intensified postoperative chemotherapy was associated with increased toxicity and did not improve event‐free survival compared to standard postoperative chemotherapy.^30^ Similar prospective studies are necessary to better understand how changes in AC may impact outcomes in GIC patients with UPR. Furthermore, beyond pathological response, the role of PET response during preoperative to direct change in chemotherapy regimen is of interest among GIC, particularly in patients with esophageal cancer undergoing concurrent chemotherapy and radiation.^31^

This study has several limitations. This was a retrospective study, which may have led to selection bias as patients who progressed or died while on NAC were excluded. Additionally, serological and radiographic data was not available for all patients. Our cohort was heterogeneous, consisting of various GI cancers, tumor stages, as well as NAC and AC regimens. Analyses by GI cancer site was limited by the modest size of the individual disease subgroups. Finally, it is possible other patient‐related factors affected a provider's decision to change AC, including declining performance status or adverse reactions to NAC regimen.

As NAC becomes more common, prospective data will be essential to understand the role of pathological response and AC decisions on patient outcomes among GIC. In our cohort of pancreatic, gastroesophageal, and colorectal cancer patients, pathologic response was associated with changes in AC, but no RFS or OS benefit was observed among those who changed AC due to UPR after NAC. Prospective interventional studies examining the role of pathological treatment response after NAC and subsequent AC decisions among specific GI cancer cohorts are needed.

## CONFLICT OF INTEREST

The authors declare there is no conflict of interest.

## AUTHOR CONTRIBUTIONS

**Huili Zhu:** Conceptualization; data curation; investigation; methodology; writing‐original draft; writing‐review & editing. **Jung‐Yi Lin:** Formal analysis; visualization; writing‐review & editing. **Umut Özbek:** Formal analysis; visualization; writing‐review & editing. **Eric Wilck:** Investigation; writing‐review & editing. **Sanders Chang:** Investigation; writing‐review & editing. **Xiuxu Chen:** Investigation; writing‐review & editing. **Stephen Ward:** Investigation; writing‐review & editing. **Noam Harpaz:** Investigation; writing‐review & editing. **Alexandros Polydorides:** Investigation; writing‐review & editing. **William Miller:** Investigation; writing‐review & editing. **Maria Isabel Fiel:** Investigation; writing‐review & editing. **Ippolito Modica:** Investigation; writing‐review & editing. **Wen Fan:** Investigation; writing‐review & editing. **Nebras Zeizafoun:** Investigation; writing‐review & editing.

## ETHICAL STATEMENT

This study was approved by the Institutional Review Board at Mount Sinai School of Medicine. Informed consent was waived because of the retrospective design of the study.

## Supporting information


**Figure S1** The median 2‐ and 5‐year recurrence‐free survival and overall survival among pancreatic, gastroesophageal, and pancreatic cancer patients.

**Table S1** Chemotherapy Regimens
**Table S2**: Univariate Analysis of Factors Associated with Recurrence‐Free after NAC and Surgery among GI Cohorts
**Table S3**: Univariate Analysis of Factors Associated with Overall Survival after NAC and Surgery among GI Cohorts

## Data Availability

The data that support the findings of this study are available from the corresponding author upon reasonable request.
