# Supplementary figures and images for: Impact of pathological response after neoadjuvant chemotherapy on adjuvant therapy decisions and patient outcomes in gastrointestinal cancers
Source: Cancer Rep (Hoboken). 2021 May 25;4(6):e1412. doi: 10.1002/cnr2.1412 (PMC8714550; doi:10.1002/cnr2.1412)

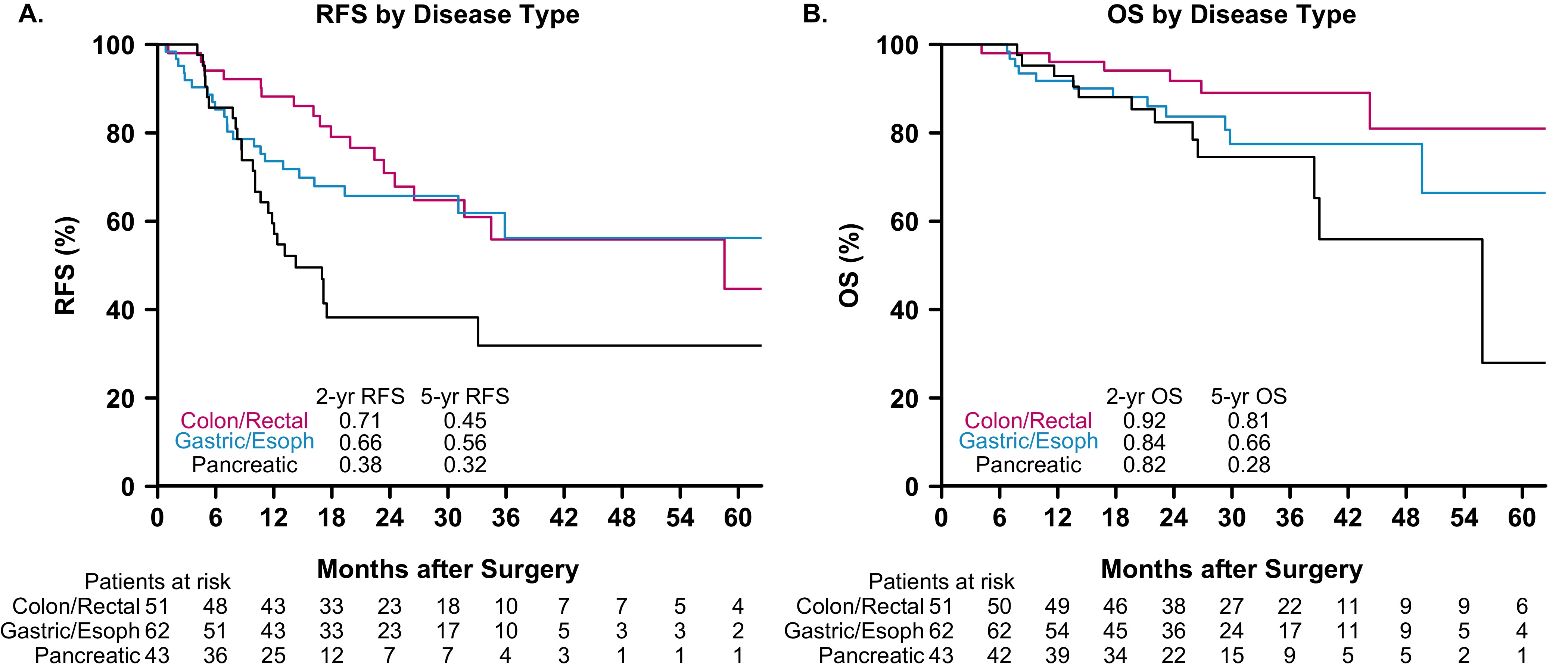

Supplement: Supplementary file 1 — Figure S1 The median 2‐ and 5‐year recurrence‐free survival and overall survival among pancreatic, gastroesophageal, and pancreatic cancer patients. [file CNR2-4-e1412-s001.jpg]
