# Supplementary material for: Impact of pathological response after neoadjuvant chemotherapy on adjuvant therapy decisions and patient outcomes in gastrointestinal cancers
Source: Cancer Rep (Hoboken). 2021 May 25;4(6):e1412. doi: 10.1002/cnr2.1412 (PMC8714550; doi:10.1002/cnr2.1412)
Supplement: Supplementary file 2 — Table S1 Chemotherapy Regimens Table S2: Univariate Analysis of Factors Associated with Recurrence‐Free after NAC and Surgery among GI Cohorts Table S3: Univariate Analysis of Factors Associated with Overall Survival after NAC and Surgery among GI Cohorts [file CNR2-4-e1412-s002.docx]

**Supplementary Table 1: Chemotherapy Regimens**

| **Tumor Site** | **Neoadjuvant Regimens (n)** | **Adjuvant Regimens (n)** |
| --- | --- | --- |
| **Pancreatic** | FOLFIRINOX (24)  Gemcitabine/nab-paclitaxel (14)  Combination or other (5) | FOLFIRINOX (7)  Gemcitabine/nab-paclitaxel (15)  Gemcitabine/capecitabine (9)  Combination or other (3) |
| **Gastroesophageal** | ECF/EOX (1)  5-FU +/- oxaliplatin (22)  Carboplatin/paclitaxel (4)  FLOT (7)  DCF (5)  Combination or other (5) | ECF/EOX (7)  5-FU +/- oxaliplatin (22)  FLOT (4)  DCF (2)  Combination or other (1) |
| **Colorectal** | 5-FU or capecitabine-based regimen (51) | 5-FU or capecitabine-based regimen (39) |

*DCF: docetaxel, cisplatin, fluorouracil; ECF: epirubicin, cisplatin, fluorouracil; EOX: epirubicin, oxaliplatin, capecitabine; FOLFIRINOX: folinic acid, fluorouracil, irinotecan, oxaliplatin; FLOT: fluorouracil, folinic acid, oxaliplatin, docetaxel; 5-FU: fluorouracil*

**Supplementary Table 2: Univariate Analysis of Factors Associated with Recurrence-Free after NAC and Surgery among GI Cohorts**

|  | **Colorectal** | | **Gastroesophageal** | | **Pancreatic** | |  |
| --- | --- | --- | --- | --- | --- | --- | --- |
| **Variable** | HR (95% CI) | P | HR (95% CI) | P | HR (95% CI) | P |  |
| FPR (CAP 0-1) | 0.30 (0.07 - 1.31) | 0.108 | 0.17 (0.02-1.26) | 0.083 | 0.35 (0.08-1.48) | 0.153 |  |
| CAP score 3 vs. 0-2 | 1.09 (0.43-2.78) | 0.856 | 3.73 (1.37-10.15) | 0.010 | 1.86 (0.86-4.04) | 0.116 |  |
| Partial or complete radiographic response | 1.91 (0.64-5.75) | 0.249 | 1.03 (0.30-3.55) | 0.964 | 0.81 (0.37-1.80) | 0.607 |  |
| ≥50% tumor marker response | 0.69 (0.25-1.89) | 0.466 | 3.80 (0.73-19.7) | 0.112 | 0.83 (0.35-1.95) | 0.665 |  |
| Clinical stage (4 vs. 1-3) | 1.87 (0.54-6.47) | 0.326 | 2.68 (0.98-7.32) | 0.054 | -^a^ | -^a^ |  |
| Pathological Stage (4 vs. 1-3 | 2.23 (0.73-6.8) | 0.158 | 9.17 (3.14-26.83) | <0.001 | 40.50 (2.53-647.48) | 0.009 |  |
| Positive surgical margins | 4.25 (1.18-15.30) | 0.027 | 5.98 (2.10-17.04) | <0.001 | 3.56 (1.17-10.83) | 0.025 |  |
| Received AC | 4.78 (1.50-15.19) | 0.008 | 1.11 (0.26-4.74) | 0.893 | 0.25 (0.25, 2.10) | 0.549 |  |
| Change of AC |  |  |  |  |  |  |  |
| Yes vs. no | 2.10 (0.7-6.26) | 0.184 | 2.56 (0.88-7.45) | 0.085 | 1.34 (0.54-3.34) | 0.523 |  |
| No AC vs. no change | 2.02 (0.61-6.63) | 0.248 | 1.67 (0.63-4.46) | 0.306 | 1.42 (0.45-4.49) | 0.550 |  |

^a^ There were no pancreatic patients with clinical stage 4 disease so the univariable model was not fitted

**Supplementary Table 3: Univariate Analysis of Factors Associated with Overall Survival after NAC and Surgery among GI Cohorts**

|  | **Colorectal** | | **Gastroesophageal** | | **Pancreatic** | |  |
| --- | --- | --- | --- | --- | --- | --- | --- |
| **Variable** | HR (95% CI) | P | HR (95% CI) | P | HR (95% CI) | P |  |
| FPR (CAP 0-1) | -^a^ | -^a^ | 0.33 (0.04-2.58) | 0.292 | -^a^ | -^a^ |  |
| CAP score 3 vs. 0-2 | 1.52 (0.31-7.53) | 0.61 | 11.67 (1.49-91.41) | 0.019 | 0.93 (0.29-2.96) | 0.903 |  |
| Partial or complete radiographic response | 0.57 (0.10-3.43) | 0.54 | 1.63 (0.23-11.60) | 0.625 | 1.20 (0.36-4.01) | 0.765 |  |
| ≥50% tumor marker response | 0.19 (0.04-1.06) | 0.059 | 1.58 (0.17-14.30) | 0.686 | 0.67 (0.16-2.86) | 0.589 |  |
| Clinical stage (4 vs. 1-3) | 1.62 (0.19-13.89) | 0.660 | 3.97 (1.16-13.58) | 0.028 | -^b^ | -^b^ |  |
| Pathological Stage (4 vs. 1-3 | -^c^ | -^c^ | 12.69 (3.50-44.10) | <0.001 | -^d^ | -^d^ |  |
| Positive surgical margins | 25.3 (4.10-156.8) | 0.001 | 6.95 (1.70-28.36) | 0.007 | 1.13 (0.14-9.07) | 0.905 |  |
| Received AC | 1.61 (0.19-13.82) | 0.665 | - ^e^ | -^e^ | 0.70 (0.15-3.32) | 0.654 |  |
| Change of AC |  |  |  |  |  |  |  |
| Yes vs. no | 3.80 (0.34-42.35) | 0.278 | 2.91 (0.46-18.43) | 0.256 | 0.87 (0.24-3.14) | 0.831 |  |
| No AC vs. no change | 7.43 (0.77-71.85) | 0.083 | 4.66 (1.17-18.54) | 0.029 | 0.54 (0.09-3.12) | 0.489 |  |

^a^There were no events in patients with CAP response so the univariable model was not fitted

^b^There were no pancreatic patients with clinical stage 4 disease so the univariable model was not fitted

^c^ There were no events in patients with pathological stage 1-3 colorectal cancer so the univariable model was not fitted

^d^ There was only one patient with pathological stage 4 pancreatic cancer so the univariable model was not fitted

^e^There were no events in gastroesophageal cancer patients receiving adjuvant therapy so the univariable model was not fitted
